# Reversibility of Endoplasmic Reticulum Stress Markers During Long-Term Glucose Starvation in Astrocytes

**DOI:** 10.1007/s12031-024-02223-5

**Published:** 2024-05-16

**Authors:** Clara Voelz, Lena E. M. Schaack, Vanessa Kogel, Cordian Beyer, Jochen Seitz, Stefanie Trinh

**Affiliations:** 1 Institute of Neuroanatomy, RWTH Aachen University, Aachen, Germany; 2 Department of Child and Adolescent Psychiatry, Psychosomatics and Psychotherapy, University of Duisburg-Essen, Essen, Germany

**Keywords:** Astrocytes, Endoplasmic reticulum stress, Unfolded protein response, Glucose starvation, cGAS-STING pathway, Anorexia nervosa

## Abstract

Previous studies have demonstrated a brain volume decrease linked to long-term starvation in patients with anorexia nervosa (AN). Food intake is critically diminished in this disorder, leading to one of the highest mortality rates within the psychiatric disease spectrum. As reported in animal models, astrocytes seem to be the most affected cell type in AN. In a recently established primary cell culture model, an elevated unfolded protein response (UPR) was observed in long-term glucose semi-starved astrocytes. A well-functioning protein machinery is essential for every cell, and prolonged UPR will lead to cell death. As a nucleic acid stress-sensing pathway with the activator located in the endoplasmic reticulum, the regulation of the cGAS-STING pathway (cyclic GMP-AMP synthase/stimulator of interferon genes) was additionally investigated in the starvation context. In the current study, a glucose semi-starvation protocol of 15 days, during which cells were supplied with 2 mM glucose in the medium, was prolonged with an additional 6-day long recovery period. Our findings showed that increased UPR mRNA expression was reversible after re-establishing the standard glucose concentration of 25 mM. Furthermore, we were able to verify the presence of cGAS and STING in astrocytes with a characteristic presence of cGAS in the astrocyte nucleus during starvation. A correlation between STING and the glial fibrillary acidic protein (GFAP) could be established, hinting at a conditional presence of STING with a specific astrocyte phenotype.

**Graphical Abstract:**

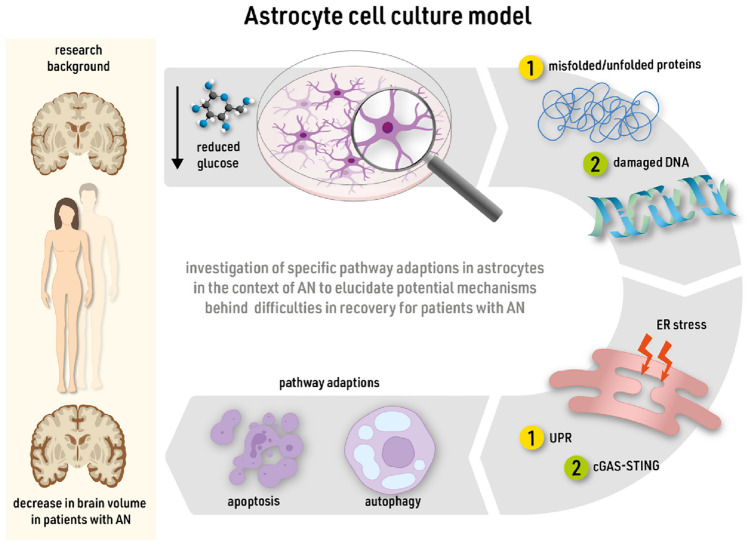

**Supplementary Information:**

The online version contains supplementary material available at 10.1007/s12031-024-02223-5.

## Introduction

Anorexia nervosa (AN) is a severe eating disorder characterized by excessive body weight loss due to limited calorie intake. It presents with one of the highest mortality rates and insufficient recovery rates. Aetiology is complex with an involvement of genetic risk factors, and pathophysiology includes psychiatric and metabolic components (Neale & Hudson 2020). Changes are found on the hormonal level (Milos & Hebebrand 2019), as well as in an elevation of inflammatory signals (Specht et al. 2022). Cognitive impairments (Berchio et al. 2023; Seitz et al. 2021) and psychiatric comorbidities such as anxiety, depression, and obsessive-compulsive behaviours (Pruccoli et al. 2023) can occur. In patients with AN, the brain volume decreases due to starvation (Bahnsen et al. 2022; Seitz et al. 2018; Walton et al. 2022). The human brain consists of different cell types with astrocytes as the most abundant. Their functions are versatile: synapse formation, involvement in the blood-brain-barrier, and the metabolic supply of neurons (Allen and Lyons 2018). In animal models for AN, in particular, the astrocyte cell population was affected during the starvation. A decrease in cell count and cell density with a reduced glial fibrillary acidic protein (GFAP) gene expression was detected, potentially due to reduced proliferation rates (Frintrop et al. 2019; Hurley et al. 2022; Reyes-Ortega et al. 2022).

To study the cellular pathophysiology of AN, we established a semi-starvation protocol for primary astrocytes (Kogel et al. 2021). In this model, astrocytes were cultured with reduced glucose concentration in the cell culture medium. They showed a heightened unfolded protein response (UPR) without increased cell mortality. Additionally, an increased expression of pro-inflammatory genes, and a genetic and morphological shift to an activated A1-phenotype were observed. Therefore, the semi-starvation model for astrocytes proved to be a useful tool to look deeper into astrocytic implications in the context of glucose reduction and brain-related alterations in AN.

**Fig. 1:**
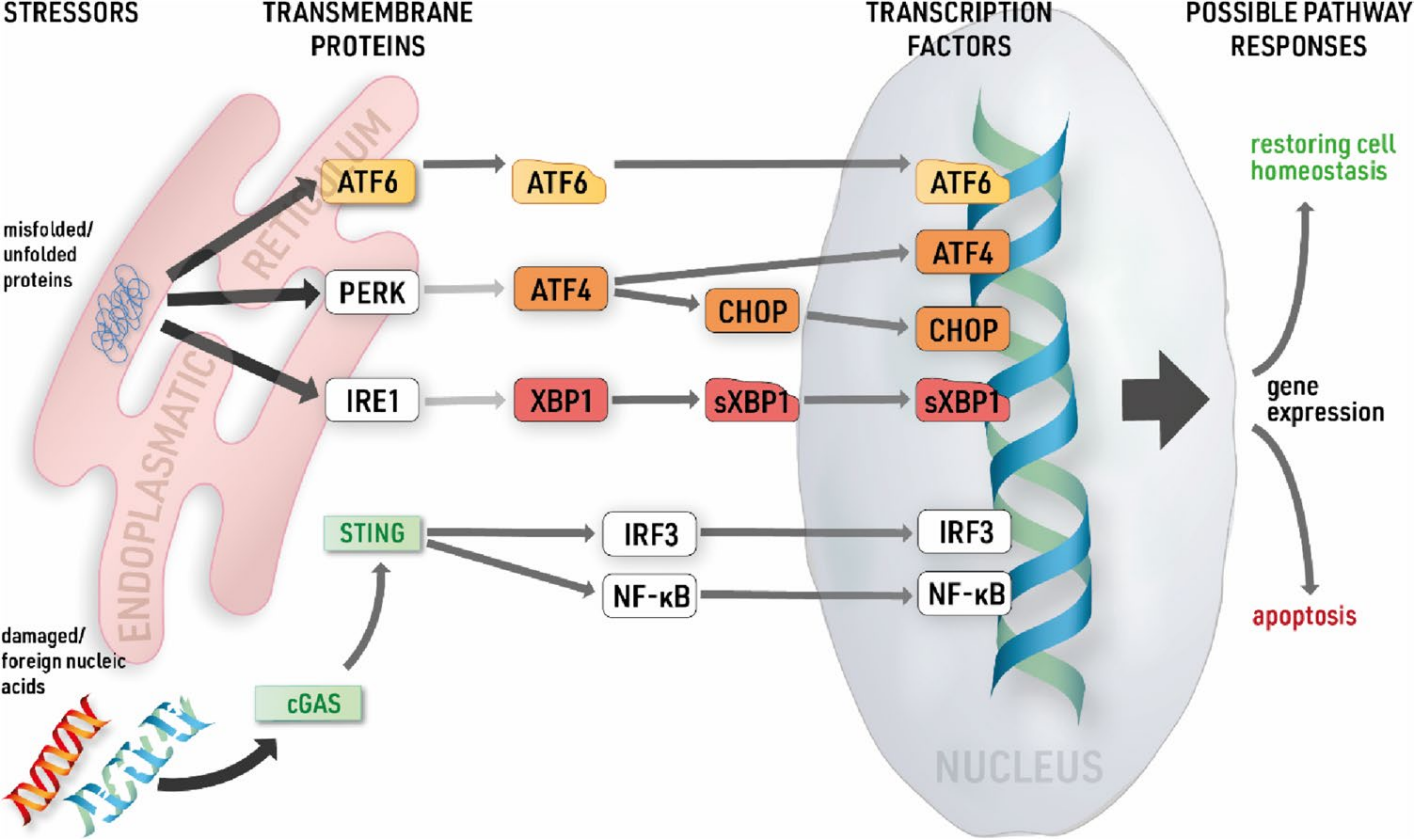
**Pathways of endoplasmic reticulum (ER) stress.** Simplified overview of different pathways involved in ER stress and subsequent UPR. White boxes indicate pathway proteins not under investigation in the present study. Sensors of protein or nucleic acid stress can be found as pathway initiators in the ER membrane. Proteins can undergo splicing or cleavage to be activated, as indicated by a change of box shape. Pathway activation will lead to expression of transcription factors that initiate various pathway responses to restore cell homeostasis or lead to apoptosis. ATF4/6, activating transcription factor 4/6; PERK, protein kinase RNA-like ER kinase; IRE1, Inositol-requiring enzyme type 1; XBP1, x-box binding protein 1; sXBP1, spliced x-box binding protein 1; CHOP, C/EBP homologous protein; IRF3, interferon regulatory factor 3; NF-κB, nuclear factor “kappa-light-chain-enhancer” of activated B cells; cGAS, cyclic GMP-AMP synthase; STING, stimulator of interferon genes

Accumulation of unfolded and misfolded proteins in the endoplasmic reticulum (ER) leads to an ER stress response and could activate different pathways of the UPR (Kaufman 2002). The UPR is a cellular adaption to restore cell homeostasis but can also lead to autophagy and apoptosis (Hetz 2012; Chipurupalli et al. 2021). There are three major UPR pathways involving the following transcription factors: X-box binding protein 1 (XBP1), activating transcription factor 4 (ATF4), C/EBP homologous protein (CHOP), and activating transcription factor 6 (ATF6) (Chipurupalli et al. 2021; Frakes and Dillin 2017; Hetz 2012) (Fig. 1). These genes had a significantly increased expression during long-term glucose semi-starvation in astrocytes (Kogel et al. 2021). Between the individual transcription factors, an interconnected activation is possible, though it should be noted that a cross-linked activation does not necessarily lead to the activation of another factor. The pathways offer a variety of interactions, depending on specific regulations. The activation of CHOP for example mediates pro-apoptotic gene induction (Rozpedek et al. 2016). Another intracellular pathway involved in sensing and mediating ER stress is the pathway of cGAS (cyclic GMP-AMP synthase) and STING (stimulator of interferon genes). The cGAS-STING pathway is known for its nucleic acid-sensing ability in the cytoplasm, mediated through cGAS (Motwani et al. 2019; Murthy et al. 2020). cGAS in turn activates STING through a second messenger. STING, located in the membrane of the ER, further activates downstream responses and initiates the activation of transcription factors.

As we have previously studied the effects of long-term glucose semi-starvation on astrocytes, this study aims to investigate the reversibility of starvation effects. After the semi-starvation period, the initial pre-experimental glucose concentration is reintroduced. Furthermore, the involvement of the cGAS-STING pathway in the cellular response to glucose semi-starvation will be elucidated. Astrocytes fulfil multiple functions within the brain and take a crucial part in supporting neurons. Therefore, impaired astrocytic function can lead to disturbances in neuronal function which in turn might contribute to cognitive impairments in patients with AN (Reville et al. 2016).

## Methods

### Primary Astrocyte Cell Culture

Wistar rat pups (RjHan:WI; Janvier Labs, Hannover, Germany) were used for cell cultures. Animals were kept according to the recommendations of the Federation of European Laboratory Associations (FELASA). Pups were decapitated, and brains were removed. In ice-cold HEPES buffer, the hemispheres were separated, and meninges, diencephalon, and mesencephalon were removed. Tissue was transferred to 10 mL Dulbecco’s modified Eagle medium (DMEM, Thermo Fisher Scientific, MA, USA) supplemented with 10% foetal calf serum (FCS; Thermo Fisher Scientific, MA, USA) and 50 U/mL penicillin and 50 µg/mL streptomycin (Thermo Fisher Scientific, MA, USA). Brain tissue was mechanically homogenized with 10 mL and 1 mL pipette tips and strained through a 70-µm cell strainer. After centrifugation, the supernatant was discarded, and cells resuspended in DMEM with 10% FCS. Cells were cultured in 75 cm^2^ cell culture flasks pre-coated with poly-L-ornithine (PLO; Sigma-Aldrich, MO, USA). Flasks were kept at 37 °C and 5% CO_2_. After the preparation, flasks were not moved for 4 days to assure cell adherence to the flask surface. Afterwards, medium was changed twice a week and cells were passaged 2 times (1:2–1:3) until the final passage during which cells were seeded for experiments to the corresponding PLO-coated plates. Before the first passage, non-adherent cell types were eliminated by placing the cell culture flasks in an orbital shaker for 2 h at 37 °C, aspirating the medium and adding new medium to the remaining adherent cells. Astrocyte purity was determined through regular extractions and counting of GFAP-positive cells after immunofluorescence staining to assure an astrocyte purity of 95% or higher.

### Experimental Setup

**Fig. 2:**
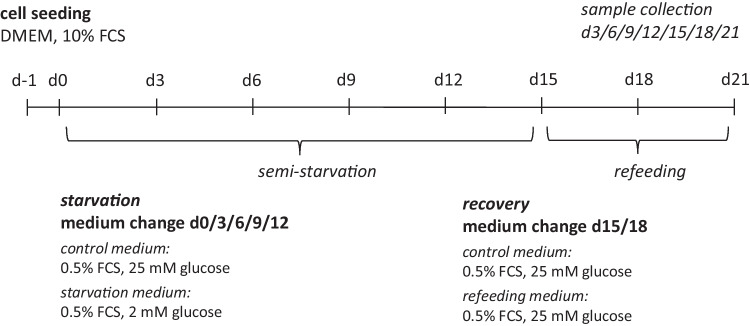
**Experimental study set-up.** The investigated cells were primary rat cortical astrocytes. After harvesting, cells were proliferated for several weeks. Cells were seeded for experiments one day before experimental begin. The experimental protocol had a duration of 21 days with medium changed and samples taken every 3 days. In the semi-starvation period from day 0 to day 15, cells in the starvation condition were semi-starved with 2 mM glucose. In the recovery period from day 15 to day 21, cells from the starvation period were cultured with 25 mM glucose. The control condition was continuously cultured with 25 mM glucose. All conditions were cultured with 0.5% FCS. FCS, fetal calf serum; d, day

Experimental treatment started 1 day after seeding the cells in standard DMEM (10% FCS). A semi-starvation protocol was previously established to provoke cell stress with a limited amount of cell death (Kogel et al. 2021). In the semi-starvation condition, cells were cultured in medium with 2 mM glucose, while the control condition was treated with a standard amount of 25 mM glucose. In both conditions, FCS was reduced to 0.5% to induce cell cycle synchronization. For the experimental medium, glucose was added to a glucose-free DMEM (Thermo Fisher Scientific, MA, USA) and then sterile filtered. Cells were kept in starvation medium for 15 days. Medium change and sample collection were performed every 3 days (Fig. 2). To test for reversibility of previously observed effects with 2 mM glucose, a recovery period of 6 days was added to the present study. During the recovery period, starting at day 15, both groups were supplied with 25 mM glucose medium.

### Cell Viability and Cytotoxicity Analysis

Cells were seeded with eight technical replicates on PLO-coated black 96-well plates with 15,000 cells per well. CellTiter-Blue^®^ Cell Viability (CTB) Assay (Promega, WI, USA, G8081) was used to determine the metabolic activity of the cells. Two biological replicates were employed for this study. According to the manufacturer’s instructions, at the designated sample collection time points (Fig. 2), CellTiter-Blue^®^ reagent was added to the cells and incubated for 2 h at 37 °C. The assay is based on the conversion of the redox dye resazurin into resorufin, a fluorescent end-product. The resulting fluorescence intensity (excitation: 560 nm, emission: 590 nm) was recorded with a Tecan infinite M200 plate reader and processed with the i-control 3.4.2.0 software (Tecan, Männedorf, Switzerland). The CytoTox 96^®^ Non-Radioactive Cytotoxicity (LDH) Assay (Promega, WI, USA, G1782) was used to determine cell cytotoxicity. According to the manufacturer’s instructions, at the designated sample collection time points, CytoTox 96^®^ working solution was added to wells and incubated for 30 min at room temperature. The assay is based on the quantification of extracellular LDH through the directly proportional formation of a dye. Absorbance was recorded at 490 nm with a Tecan infinite M200 plate reader and processed with the i-control 3.4.2.0 software (Tecan, Männedorf, Switzerland).

### Immunofluorescence Staining

Cells were seeded on PLO-coated glass coverslips in 24-well plates with 50,000 cells per well. Cells were fixated with 3.7% paraformaldehyde (Carl Roth Karlsruhe, Germany), and cell membranes were permeabilized with 0.2% Triton X-100 (Carl Roth Karlsruhe, Germany). Unspecific binding was blocked with blocking buffer (1% BSA and 2% FCS in PBS) for 1 h. Cells were incubated with respective antibodies in blocking buffer overnight (Table 1). Then, cells were incubated with the respective secondary antibodies for 1 h and counterstained with Hoechst 33342 solution (1:10,000, Thermo Fisher Scientific, MA, USA) for 5 min. Coverslips were mounted with Epredia™ Shandon™ Immu-Mount™ (Thermo Fisher Scientific, MA, USA) onto glass slides. For picture acquisition, cells were imaged with a DMI6000 B fluorescence microscope (Leica Biosystems, Wetzlar, Germany).

**Table 1:** Primary and secondary antibodies used for immunofluorescence staining

Primary antibody			Secondary antibody		
**GFAP** goat	**Abcam** ab107159	1:1000	Alexa Fluor goat anti-chicken 594	**Life Technologies** A11042	1:500
**GGAS** Rabbit	**Thermo Fisher Invitrogen** PA5-76-367	1:200	Alexa Fluor donkey anti-rabbit 488	**Life Technologies** A21206	1:500
**STING** Rabbit	**Abcam** ab227704	1:100	Alexa Fluor donkey anti-rabbit 488	**Life Technologies** A21206	1:500

### RNA Extraction and Analysis

For RNA isolation and gene expression studies, cells were seeded on PLO-coated 6-well plates with 400,000 cells per well. Four biological replicates were employed in this study. RNA was extracted by removing cell culture medium from wells and solving cells in 600 µL RNA-Solv^®^ Reagent (Omega Bio-tek, Inc., GA, USA). The isolation is based on a phenol-chloroform phase separation, whereby the upper aqueous phase is used to extract total RNA content. RNA concentration and purity were measured with a NanoDrop 1000 spectrometer (Thermo Fisher Scientific, MA, USA). Reverse transcription to cDNA was performed with a M-MLV Reverse Transcriptase (Thermo Fisher Scientific, MA, USA) with random primer (Thermo Fisher Scientific, MA, USA) according to the manufacturer’s instructions. Semi-quantitative real-time PCRs (qRT-PCR) were performed with an AceQ SYBR Green qPCR Master Mix (Nanjing Vazyme Biotech Co, Nanjing, China) with the listed target primers (Table 2) and measured with a CFX Connect™ Real-Time system (BioRad, Feldkirchen, Germany). Data were analysed through the ΔΔCt-method. For each sample, values of the gene of interest were normalized to the relative quantity of the reference gene Cyclophilin A.

**Table 2:** List of primers used in this study. bp = basepairs, AT = annealing temperature

**ATF4**	129 bp, AT: 62 °C	**IRF3**	75 bp, AT: 65 °C
Sense	GCTCTTCACGAAACCCAGCA	Sense	CTCCCCTGGCTCAGGAACT
Antisense	CCAACACTTCGCTGTTCAGGA	Antisense	CCAAGGCAAAATCAGCGGTT
**ATF6**	124 bp, AT: 60 °C	**Psmb8**	128 bp, AT: 64 °C
Sense	TTCTTCAACTCAGCACGTTCC	Sense	CGGGACACTACAGTTTCTCCGT
Antisense	AGGCTTCTCTTCCTTCAGTGG	Antisense	GCCGTGCGCCATTTCAATCT
**cGAS**	75 bp, AT: 65 °C	**S100a10**	100 bp, AT: 60 °C
Sense	GGCCGAGACGGTGAATAAAGT	Sense	CCCTCTGGCTGTGGACAAAAT
Antisense	ACGCCTTTGAACTCGGACTC	Antisense	AATGATGAGCCCCGCCACTA
**CHOP**	108 bp, AT: 65 °C	**STING1**	127 bp, AT: 65°C
Sense	TGTTGAAGATGAGCGGGTGG	Sense	GACTGCTGTCTGCCCTTTGA
Antisense	GCTTTCAGGTGTGGTGGTGT	Antisense	GGATGGATGCAGGTTGGAGT
**Cyclophilin A**	196 bp, AT: 65 °C	**sXBP1**	169 bp, AT: 60 °C
Sense	GGCAAATGCTGGACCAAACAC	Sense	TGCTGAGTCCGCAGCAGGTG
Antisense	TTAGAGTTGTCCACAGTCGGAGATG	Antisense	GCTGGCAGACTCTGGGGAAG
**IFNB1**	132 bp, AT: 65 °C	**tXBP1**	147 bp, AT: 60 °C
Sense	CGACTACAAGCAGCTCCAGT	Sense	GAAAGAAAGCCCGGATGAGC
Antisense	GGGTGCATCACCTCCATAGG	Antisense	TCCCCAAGCGTGTCCTTAAC

### Protein Extraction and Analysis

Cells were seeded on PLO-coated 10 cm dishes with two million cells per dish. Four biological replicates were employed in this study. After treatment, protein isolation was started by detaching cells with trypsin. Medium was added for neutralisation. Cell suspension was collected and centrifugated at 4 °C. The cell pellets were collected and resuspended in 50 µL RIPA buffer. Protein concentration was measured with the Pierce™ BCA Protein Assay Kit (Thermo Fisher Scientific, MA, USA) which was used according to manufacturer’s instructions. Protein concentration was measured with a Tecan infinite M200 plate reader and processed with the i-control 3.4.2.0 software (Tecan, Männedorf, Switzerland). For the SDS-Page, 12% (v/v) SDS-gels were used. Samples were adjusted to 20 µg protein per 20 µL reaction volume with RIPA buffer and Laemmli buffer. The Spectra Multicolor Broad Range Protein Ladder (Thermo Fisher Scientific, MA, USA) was used for band size reference. For blotting in a semi-dry setup, gels were transferred to PVDF Western blotting membranes (Roche Holding AG, Basel, Switzerland). Membranes were blocked for 1 h in 5% milk or 5% BSA depending on the solvent of the primary antibody (see Table 3). Membranes were incubated overnight with the corresponding primary antibodies. Afterwards, they were incubated with a secondary antibody for 2 h. Membranes were developed with an ECL chemiluminescence kit (Thermo Fisher Scientific, MA, USA). For evaluation of the individual protein signal, a densitometric analysis of bands was performed and later normalized to the GAPDH reference bands using the ImageJ software (NIH, Bethesda, MD, USA). For full-length western blots, please refer to Supplementary Fig. 1–3.

**Table 3:** Primary and secondary antibodies used for Western blot

Primary antibody			Secondary antibody		
**GAPDH** Rabbit	**Santa Cruz** sc-25778	1:5,000 (in 5% milk)	IgG (H+L)-HRP (Bio-Rad)	**Bio-Rad** 170-6515	1:5000 (in 5% milk)
**cGAS** Rabbit	**Thermo Fisher Invitrogen** PA-5-76367	1:500 (in 5% milk)	IgG (H+L)-HRP (Bio-Rad)	**Bio-Rad** 170-6515	1:5000 (in 5% milk)
**STING** Rabbit	**Abcam** ab227704	1:800 (in 5% BSA)	IgG (H+L)-HRP (Bio-Rad)	**Bio-Rad** 170-6515	1:5000 (in 5% milk)

### Statistical Analysis

Statistical analysis of experimental data was performed with GraphPad Prism 9.1.1 and IBM SPSS Statistics 22. Data are indicated as arithmetic mean±standard deviation unless stated otherwise. The statistical evaluation for semi-starvation and refeeding was divided; thus, two-way ANOVA was calculated on both conditions individually to only include two parameters in the comparison (time and treatment). Normal distribution was tested with the Shapiro-Wilk test. In case of non-normal distribution, a Box-Cox transformation with an adequate lambda was applied. When the data tested positive for normal distribution, statistical significance was tested with a two-way ANOVA test. A *p* ≤ 0.05 was considered as statistically significant. For western blot analysis, a normalization to the control group per each timepoint was performed. Therefore, a statistical evaluation was not performed.

## Results

Primary rat astrocytes were subjected to reduced glucose levels for 15 days to investigate the impact on cell metabolism and stress responses. The subsequent re-supplementation of glucose for 6 days allowed the examination of reversibility of the regulatory mechanisms during starvation.

### Metabolic Activity but not Cytotoxicity Was Affected

**Fig. 3:**
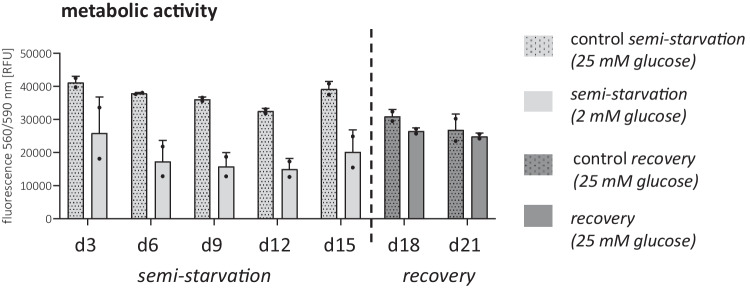
**Metabolic activity of semi-starved primary astrocytes.** Metabolic activity was assessed with a CTB assay with eight technical replicates in two independent experiments (*n* = 2). Graph depicts mean ± SD

The use of a 2 mM glucose cell culture medium led to an overall reduction in cellular metabolic activity (Fig. 3). During the starvation phase, metabolic activity decreased in the semi-starved group compared to the control. Following glucose supplementation on day 15, differences in metabolic activity between the groups diminished equalizing the fluorescence absorbance between control and experimental group. A cytotoxicity assessment showed no discernible differences among the groups at all time points (data not shown).

### Unfolded Protein Response (UPR) Was Highly Reversible in Gene Expression

**Fig. 4:**
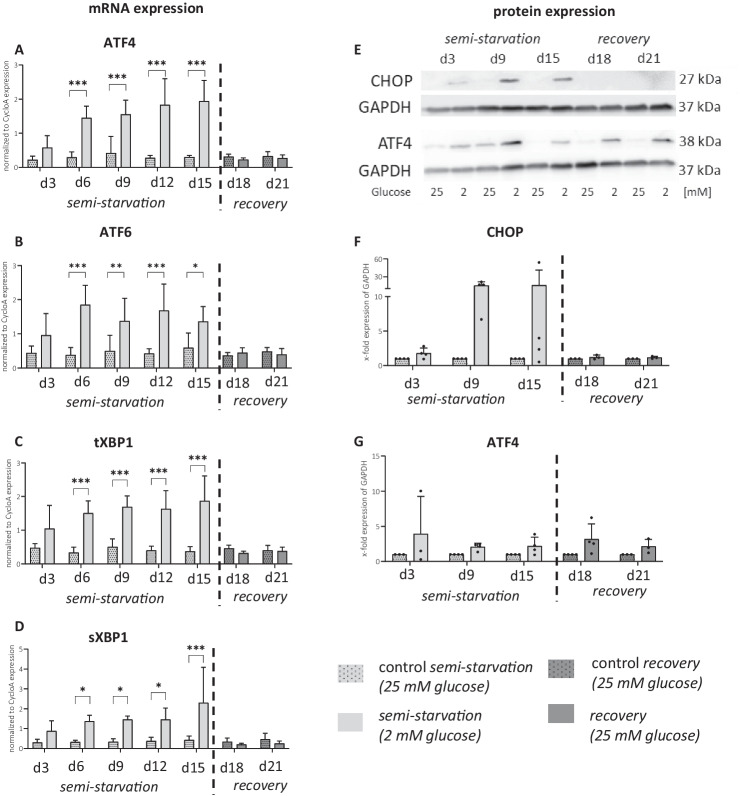
**Unfolded protein response in semi-starved astrocytes. A**, **B**, **C**, and **D** show relative mRNA expression of unfolded protein response markers from four independent experiments (*n* = 4). Graphs depict mean ± SD. Two-way ANOVA was calculated on semi-starvation period and recovery period individually. ***p ≤ 0.001, **p ≤ 0.01, *p ≤ 0.05. **E** shows protein, and **F** and **G** show relative normalized protein expression of unfolded protein response markers. Dots in graphs indicate replicates from independent experiments. Data was normalized to the control of each time point, and statistical analysis was not performed

Glucose-starved cells exhibited elevated activity in genes associated with the UPR (Fig. 4). The gene expression of all UPR transcription factors followed a similar trend. When glucose was reduced in the experimental group, the UPR gene expression immediately increased at day 3 and remained elevated over the whole time of semi-starvation. The effect reached the significant threshold at the experimental time point of day 6 (*p* < 0.001) for ATF4 (Fig. 4A), ATF6 (Fig. 4B), tXBP1 (Fig. 4C), and sXBP1 (Fig. 4D). Notably, two variants of XBP1 were studied: the total transcript (tXBP1) and the spliced form (sXBP1), which is indicative of UPR-related transcription factor activity. When glucose was re-supplemented, the effect was reversed for all UPR factors, equalizing the experimental and the control group.

On the protein expression level, the CHOP protein was only detectable in the semi-starvation condition. It was observable mainly on day 9 and day 15 in culture, but not yet on day 3, suggesting a longer pathway response time until CHOP protein formation (Fig. 4E+F). Upon glucose re-supplementation, CHOP protein levels normalized at day 18 and day 21. Protein levels of ATF4 showed a slight increase in the semi-starvation group, with no abrupt change upon glucose re-supplementation, possibly due to slower translational processes, hinting at ATF4 being more consistently expressed after glucose starvation (Fig. 4G).

### STING Gene and Protein Levels and Cellular Localization

**Fig. 5:**
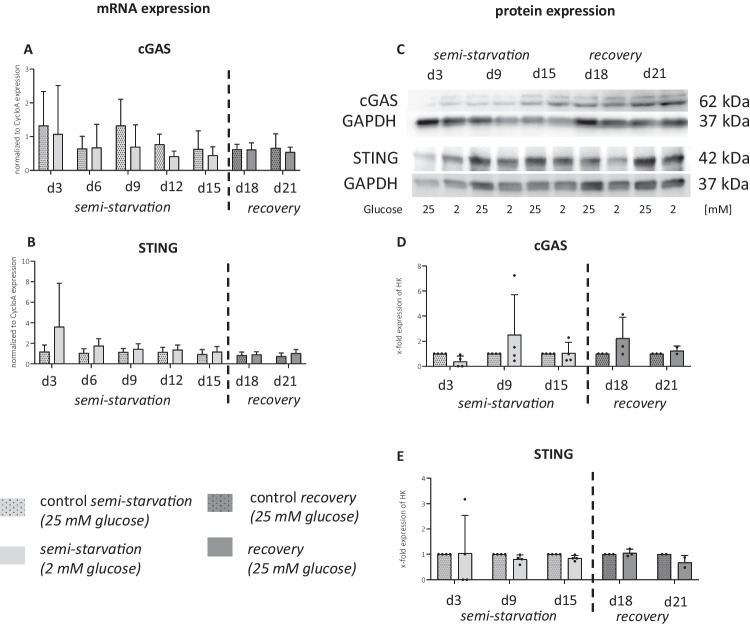
**cGAS-STING pathway in semi-starved astrocytes.** **A** and **B** show relative mRNA expression of cGAS-STING pathway markers from four independent experiments (*n* = 4). Graphs depicts mean ± SD. Two-way ANOVA was calculated on semi-starvation period and recovery period individually. No significant difference was found in the comparison between “control” and “starvation” at the indicated time points. **C** shows protein, and **D** and **E** show relative normalized protein expression of cGAS-STING pathway markers. Dots in graphs indicate replicates from independent experiments. Data was normalized to the control of each time point and statistical analysis was not performed

The STING signalling pathway, a mediator of intracellular DNA stress, showed subtle differences between the semi-starvation group and control group, without statistical significance (Fig. 5A–E). Glucose re-supplementation did not yield noticeable effects. ANOVA indicated an impact of the starvation period on gene expression of cGAS (*p* = 0.0145) and STING (*p* = 0.0012) during the initial 15 days. Within this experimental set-up, the expression of cGAS and STING seems to be unaffected in quantity.

**Fig. 6:**
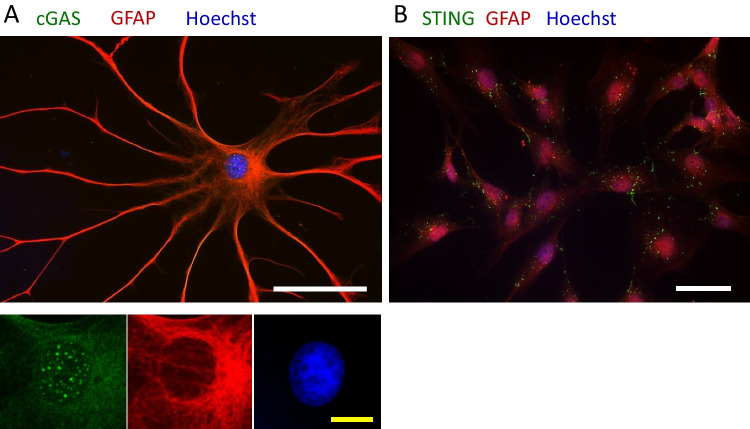
**Localization of cGAS and STING within astrocytes in glucose semi-starvation.** Exemplary pictures are shown from GFAP-positive astrocytes (in red) cultured with 2 mM glucose that were either coupled with cGAS (**A**) or STING (**B**) (in green). White scale bar is 50 µm; yellow scale bar is 10 µm

**Fig. 7:**
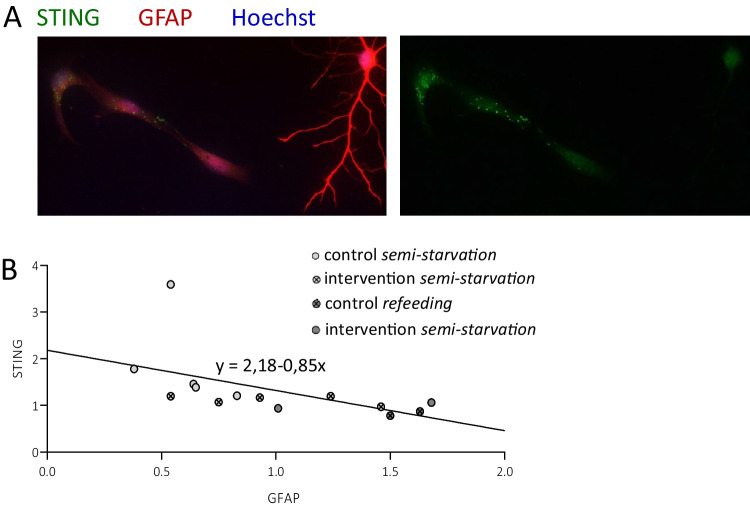
**Immunofluorescence signal of GFAP and STING**. **A** Exemplary pictures are shown from GFAP-positive astrocytes (in red) cultured with 25 mM condition glucose. Astrocytes with a strong GFAP intensity do not display STING vacuoles (in green). White scale bar is 50 µm. **B** This observation is supported by the correlation of mRNA expression of STING and GFAP, demonstrating a negative correlation (*r* =  − 0.541, *p* = 0.046). The mean value of replicates was summarized in the graph. Data was collected from four independent experiments (*n* = 4)

As quantification of cGAS and STING transcription and protein alone were not able to provide further insight into pathway regulation, we investigated other pathway aspects with their position within the cell (Fig. 6). Notably, cGAS protein accumulation within the nucleus was observed exclusively during starvation, indicating a conditional nuclear presence in glucose scarcity (Fig. 6A). STING vacuoles could be observed within the cytosol of some GFAP-positive astrocytes (Fig. 6B), but no prominent STING vacuoles were observed in astrocytes with a strong GFAP-positivity (Fig. 7). Correlation analysis of GFAP and STING gene expression data revealed a negative association (*r* = −0.541, *p* = 0.046).

## Discussion

In patients with AN, weight restoration remains the primary therapeutical approach. Even though the cognitive impairments are improving after body weight increase, follow-up measurements in recovered patients still show a reduced brain volume (Seitz et al. 2018). The astrocyte semi-starvation cell culture model was established to mimic effects of glucose reduction in the brain in disorders such as AN. Previous results demonstrated that astrocytes respond to this condition with an increase in UPR markers (Kogel et al. 2021). To test for reversibility of effects, a recovery phase was now added to the model. The recovery phase demonstrated a reversal of upregulation for most of the UPR markers upon glucose recovery. In our recovery astrocyte model, not only the UPR but also the cGAS-STING pathway was studied. Here, the starvation had no observable effect on the quantitative gene and protein expression, but the organization of structures within the cell compartments changed during starvation. This observation is the basis for new research questions regarding the influence of innate immunity response and sterile inflammation to astrocytes, mediated by the cGAS-STING pathway.

The capability of astrocytes to quickly adapt to environmental alterations, including fluctuations in nutrient availability such as glucose, has been described before (Arend et al. 2019; Giovannoni and Quintana 2020). The capacity of adaptation is owed to an array of receptors on the astrocyte membrane. The receptors process signals of neurotransmitters, neuromodulators, and other signalling molecules. The glucose transporter GLUT1 senses and transports the sugar molecules across the cell membrane along a concentration gradient (Gandhi et al. 2009). When glucose is reduced, cell metabolism is changed. There is a switch towards lactate production to further maintain energy resources for neuronal cells (Arend et al. 2019). As an adaption to glucose scarcity, astrocytes are able to maintain glycogen storages, demonstrating their metabolic flexibility (Dringen et al. 1993; Dringen and Hamprecht 1993). In another semi-starved astrocytic culture, the switch from the glycolytic glucose metabolism to a lactate-based ATP production was observed within 2 days of glucose reduction (Arend et al. 2019).

The upregulation of the UPR response is a sign of cellular stress. The outcome of the UPR controls the cellular fate. ER homeostasis and folding capacity are either restored or cells undergo apoptosis if the accumulation of unfolded and misfolded proteins is not manageable (Hetz 2012; Chipurupalli et al. 2021). The present results support the idea that the reduction of glucose leads to excessive stress that influences ER function. Glucose starvation can lead to a change in intracellular metabolism pathways that affect protein folding processes. The function of the ER is closely linked to the availability of energy and metabolic substrates like glucose. If, in the absence of glucose, less ATP is produced, protein folding cannot be performed adequately and traffic within the ER will be disrupted (Depaoli et al. 2019). The observed activation of UPR upon glucose reduction reflects a connection between glucose availability, cellular metabolism, and protein folding within the ER.

The astrocyte semi-starvation cell culture model is emulating a complex eating disorder. With the help of this model, we want to investigate the reaction of astrocytes to glucose starvation. It serves to analyse astrocyte stress, modelling a static moment in cell dynamics. Neither proliferation rate nor mortality rate was shown to be affected in the model. This enables the study of the mechanisms prior to critical events such as massive cell death. Studying pathway reversibility is crucial to determine astrocyte flexibility. Uncovering pathway mechanisms susceptible to drug intervention are needed to find approaches saving the pathological phenotype observed in patients with AN. Molecular changes that have been adaptive during starvation, but that are persisting throughout recovery, might be maladaptive and could lead to a higher probability of relapse on the systemic level. The non reversibility -of ATF4 protein upregulation in our model is to be further investigated. It should be determined if this phenotype can be rescued under a prolonged recovery period. The ATF4 mRNA expression is quick to adapt, hinting at a lack of ATF4 protein degradation. CHOP protein expression on the other hand is immediately downregulated in recovery. The persistence of ATF4 protein expression might keep astrocytes more susceptible to UPR activation. This could help enforce homeostasis by activating necessary pathways towards cell survival or towards apoptosis, depending on the degree of cellular stress.

Another stress-pathway in connection with the ER is the cGAS-STING pathway. This innate immune signalling pathway plays a crucial role in detecting cytoplasmic nucleic acids (Zhang et al. 2020). Nutrient deficiency can lead to increased DNA damage and mitochondrial damage, as the organism has a decreased ability to manage reactive oxygen species (Kaliszewska et al. 2021; Kaźmierczak-Barańska et al. 2020). cGAS can be present in the cytoplasm as well as in the nucleus where it is bound by nucleosomes (Barnett et al. 2019; Michalski et al. 2020; Zhao et al. 2020). cGAS in the cytoplasmic environment can activate the cGASSTING pathway as response to nucleic acids, but cGAS in the nucleus remains inactive. In the nucleus, nucleosomes block dsDNA binding (Michalski et al. 2020; Zhao et al. 2020), but also block DNA repair (Li et al. 2022). Accumulation of unrepaired DNA damage will lead to apoptosis. cGAS can also attach to short telomeres avoiding mitotic chromosome end-to-end fusions which would lead to further aberrant DNA occurrences (Li et al. 2022).

A characteristic presence of cGAS protein in the nucleus was observed during semi-starvation. This is demonstrating a cGAS-STING pathway regulation depending on glucose concentration. The function of nuclear cGAS in this context is to be determined. Then, a lower GFAP-positivity was linked to a higher quantity of STING protein in astrocytes. The finding was confirmed through mRNA expression levels. This is hinting towards the presence of the cGAS-STING pathway in a specific astrocytic phenotype. For both cGAS and STING activity, a change in mRNA and protein quantity might not be detectable in a heterogenous astrocyte population. Furthermore, a focus might need to be put on the modulation of different pathway proteins as to shed light on cGAS-STING pathway activation during starvation.

Further studies should also focus on the intercellular dynamics of the CNS. This could include co-culturing astrocytes with neurons. Astrocytes support neurons at several levels, as through maintaining homeostasis and providing metabolic support (Bonvento and Bolaños 2021; Durkee et al. 2021). It should be clarified how alterations of the UPR and cGAS STING pathway affect astrocytes in their supporting role and consequently affect neuronal functioning.

## Conclusion

Cell dynamics, like alterations in cell numbers and stress responses, are important regulatory mechanisms in the impaired brain of patients with AN. If we understand these processes and their implications on brain function, AN pathophysiology, and chronic manifestation of the illness could be addressed. In the present study, it was demonstrated that re-supplementing of glucose after a semi-starvation period led to a mostly instant reduction of UPR response. A focus can be put on processes that are differentially regulated during starvation and that cannot be reversed through re-supplementation of glucose. Hardship in recovery of patients with AN could potentially be due to persistent maladaptive pathway regulations. Those pathways could be targeted to facilitate and stabilize recovery.

### Supplementary Information

Below is the link to the electronic supplementary material.

Supplementary file1 (DOCX 1177 KB)

## Data Availability

Datasets generated and analyzed during this study are available from the corresponding author upon reasoned request.
