# Supplementary material for: Reversibility of Endoplasmic Reticulum Stress Markers During Long-Term Glucose Starvation in Astrocytes
Source: J Mol Neurosci. 2024 May 16;74(2):53. doi: 10.1007/s12031-024-02223-5 (PMC11096255; doi:10.1007/s12031-024-02223-5)
Supplement: Supplementary file 1 — Supplementary file1 (DOCX 1177 KB) [file 12031_2024_2223_MOESM1_ESM.docx]

**Supplementary material**


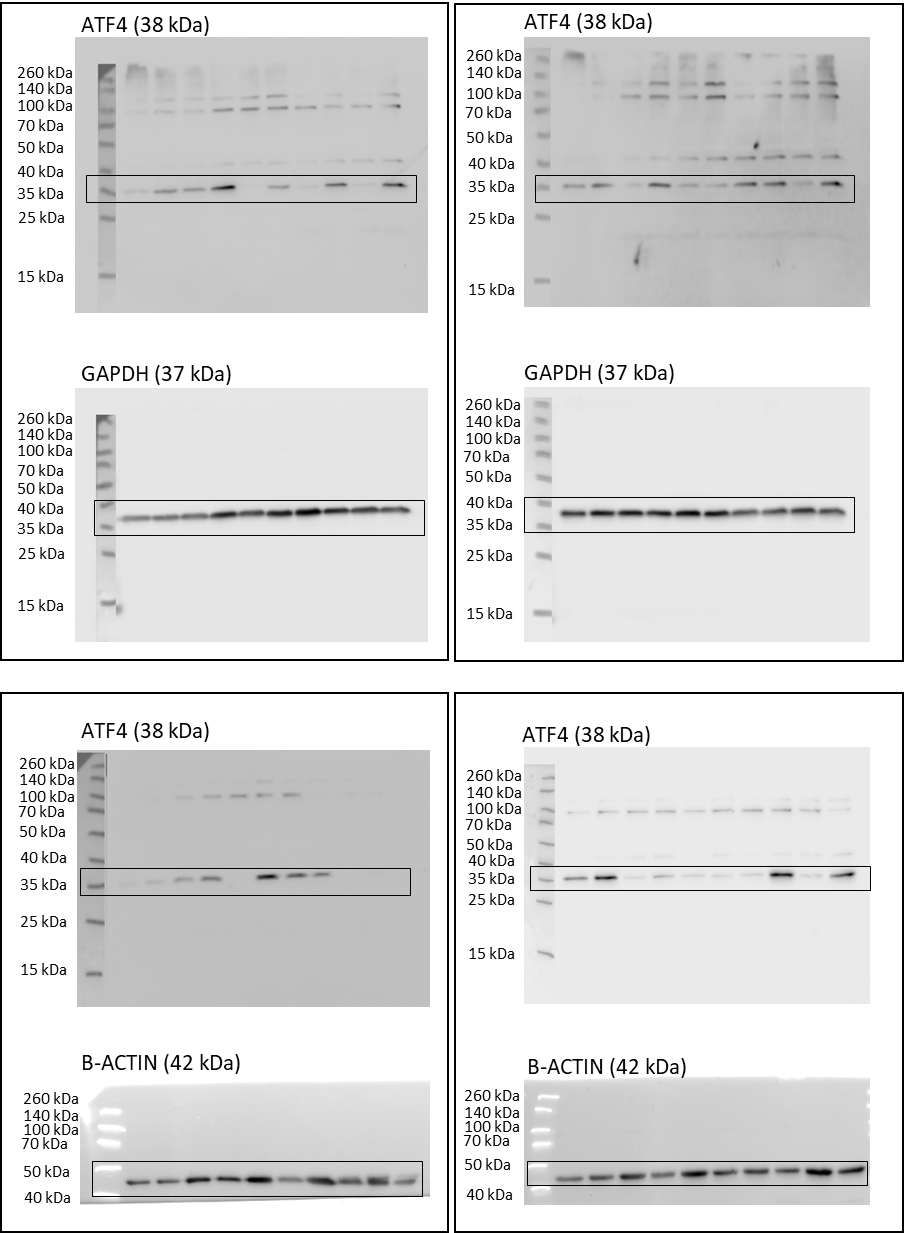

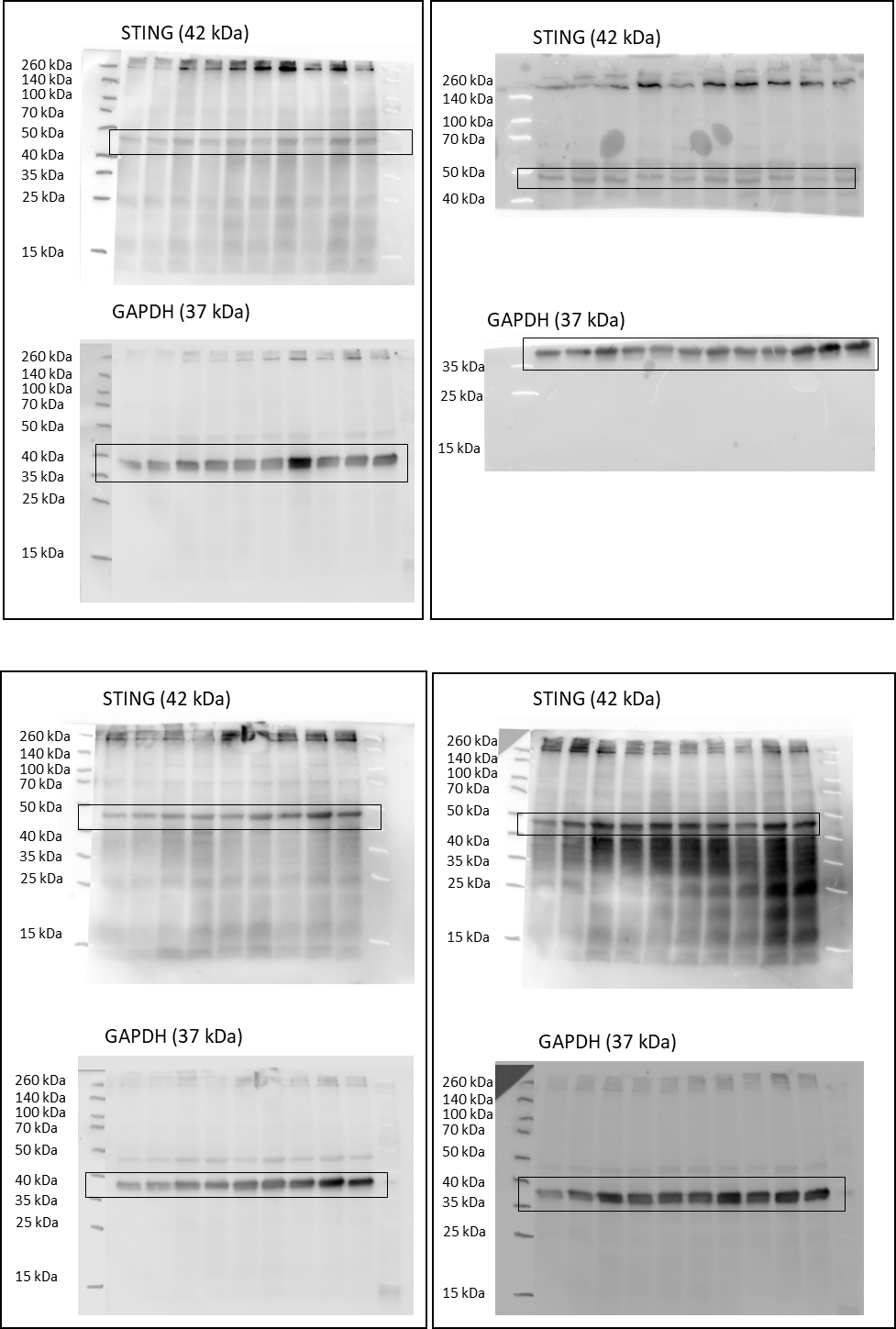


Supplementary Figure 1: Full-length western blots of ATF4, along with the associated reference protein GAPDH and B-ACTIN.

Supplementary Figure 2: Full-length western blots of STING, along with the associated reference protein GAPDH.


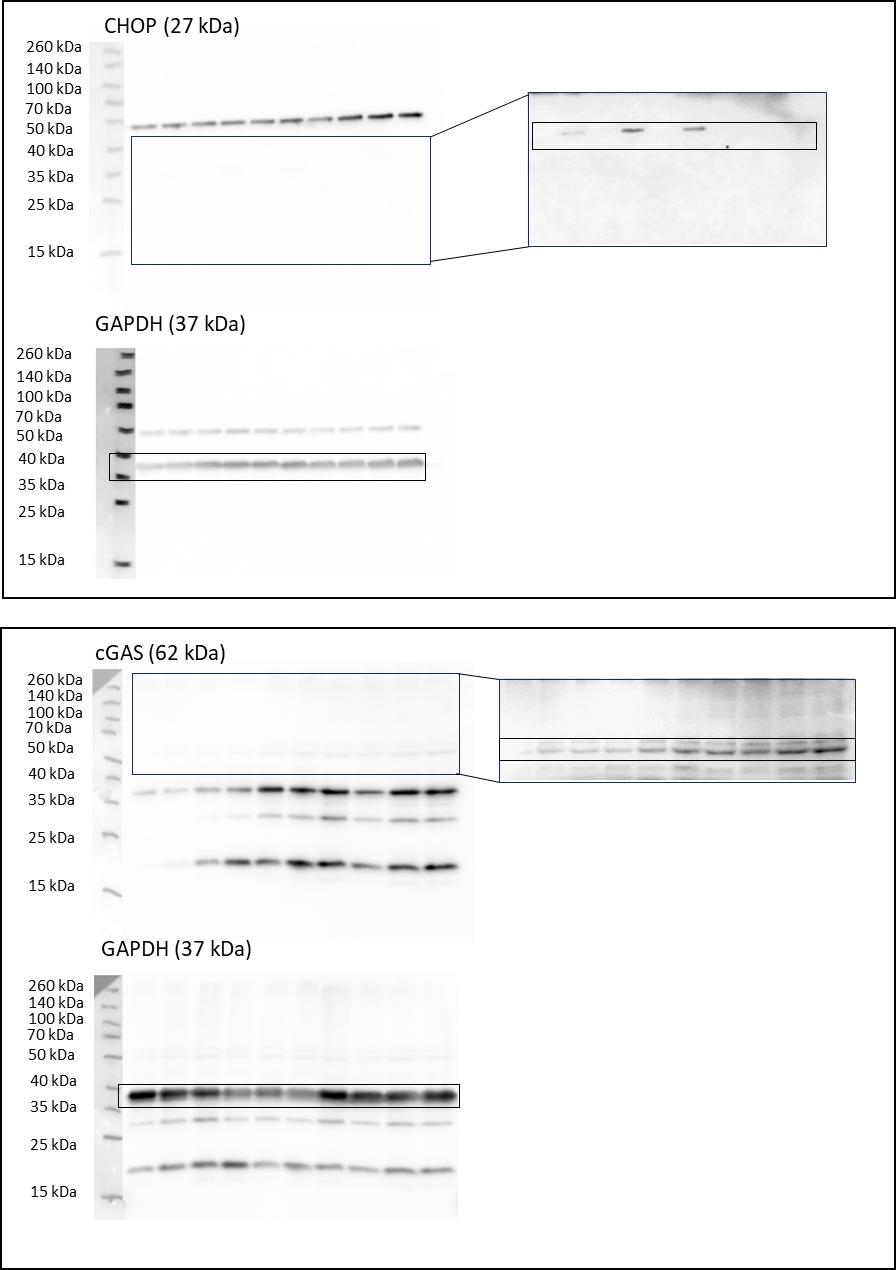


Supplementary Figure 3: Full-length western blots of CHOP and cGAS, along with the associated reference protein GAPDH. To enhance the protein signal, specific regions on the blot were inverted.
